# Effect of Dialysis Initiation Timing on Clinical Outcomes: A Propensity-Matched Analysis of a Prospective Cohort Study in Korea

**DOI:** 10.1371/journal.pone.0105532

**Published:** 2014-08-19

**Authors:** Jeonghwan Lee, Jung Nam An, Jin Ho Hwang, Yong-Lim Kim, Shin-Wook Kang, Chul Woo Yang, Nam-Ho Kim, Yun Kyu Oh, Chun Soo Lim, Yon Su Kim, Jung Pyo Lee

**Affiliations:** 1 Department of Internal Medicine, Hallym University Hangang Sacred Heart Hospital, Seoul, Korea; 2 Clinical Research Center for End Stage Renal Disease (CRC for ESRD), Daegu, Korea; 3 Department of Internal Medicine, Seoul National University Boramae Medical Center, Seoul, Korea; 4 Department of Internal Medicine, Chung-Ang University Medical Center, Seoul, Korea; 5 Department of Internal Medicine, Kyungpook National University School of Medicine, Daegu, Korea; 6 Department of Internal Medicine, Yonsei University College of Medicine, Seoul, Korea; 7 Department of Internal Medicine, The Catholic University of Korea College of Medicine, Seoul, Korea; 8 Department of Internal Medicine, Chonnam National University Medical School, Gwangju, Korea; 9 Department of Internal Medicine, Seoul National University Hospital, Seoul National University College of Medicine, Seoul, Korea; University of Sao Paulo Medical School, Brazil

## Abstract

**Background:**

Controversy persists regarding the appropriate initiation timing of renal replacement therapy for patients with end-stage renal disease. We evaluated the effect of dialysis initiation timing on clinical outcomes. Initiation times were classified according to glomerular filtration rate (GFR).

**Methods:**

We enrolled a total of 1691 adult patients who started dialysis between August 2008 and March 2013 in a multi-center, prospective cohort study at the Clinical Research Center for End Stage Renal Disease in the Republic of Korea. The patients were classified into the early-start group or the late-start group according to the mean estimated GFR value, which was 7.37 ml/min/1.73 m^2^. The primary outcome was patient survival, and the secondary outcomes were hospitalization, cardiovascular events, vascular access complications, change of dialysis modality, and peritonitis. The two groups were compared before and after matching with propensity scores.

**Results:**

Before propensity score matching, the early-start group had a poor survival rate (P<0.001). Hospitalization, cardiovascular events, vascular access complications, changes in dialysis modality, and peritonitis were not different between the groups. A total of 854 patients (427 in each group) were selected by propensity score matching. After matching, neither patient survival nor any of the other outcomes differed between groups.

**Conclusions:**

There was no clinical benefit after adjustment by propensity scores comparing early versus late initiation of dialysis.

## Introduction

Chronic kidney disease (CKD) is a major public health problem, and the number of patients requiring dialysis for end-stage renal disease (ESRD) has been increasing rapidly around the world [1]. In 2011, the prevalence of dialysis patients in the United States was 430,273 (0.2% of the general population), and the rate of ESRD cases per million population reached 1,901 [2]. Medical expenditures for patients with ESRD in the United States reached $49.3 billion in 2011, and ESRD patients accounted for 6.3% of total Medicare costs. Most cases of ESRD are complicated, with various comorbidities, and are associated with poor health outcomes. In the United States, ESRD patients were reported to have a residual life expectancy of 6.2 years [2]. According to two large population cohort studies in Canada and Taiwan, patients aged 55 years with stage 5 CKD or who were on dialysis had life expectancies of 5.6 and 12.0 years and cardiovascular mortality rates of 58% and 71%, respectively [3]–[5]. Adequate dialysis therapy can relieve the burden of painful uremic symptoms and improve overall survival [6]–[8]. Therefore, it is important to initiate dialysis therapy at the appropriate time to prevent fatal uremic complications and improve patient survival.

However, controversy persists regarding the optimal timing for dialysis initiation. There is a conventional belief that delaying dialysis until the patient's eGFR falls below 6 ml/min/1.73 m^2^ was potentially dangerous and that starting dialysis early could improve the nutritional status and survival of ESRD patients through increased uremic solute clearance, particularly in patients with diabetes or high comorbidities [9]. According to data from the United States Renal Data System (USRDS), the proportion of patients who started dialysis at an eGFR greater than 10 ml/min/1.73 m^2^ had been steadily increasing up to 2009, reaching 54% of the patient population [10]. However, several observational and meta-analysis studies reported that early initiation of dialysis is associated with certain harmful clinical outcomes [11]–[14]. To date, there has been only one prospective, randomized, controlled study (the Initiating Dialysis Early and Late Trial; IDEAL study) on dialysis initiation time and patient survival. This study reported that planned early initiation of dialysis was not associated with improvements in either survival or clinical outcomes [15]. Recent guidelines reflecting the results of the IDEAL study emphasize that the eGFR, based on serum creatinine levels, should not be the only factor used to guide dialysis initiation time and recommend that dialysis should be preferentially deferred until the development of uremic symptoms or complications [16]–[18].

Clinical outcomes and mortality rates among patients initiating dialysis are known to differ according to the demographic characteristics of race or ethnicity [19]. Asian advanced CKD patients have lower mortality and cardiovascular morbidity than Caucasians despite a faster decline in GFR and a higher incidence of renal replacement therapy [20]–[22]. However, clinical evidence on dialysis initiation time and associated clinical outcomes is insufficient among non-Caucasian populations. Although there are a few retrospective, observational studies on dialysis initiation timing among Asian ESRD patients [23]–[27], single-center designs and/or insufficient adjustments in multivariate analyses attributable to limited data collection from retrospective designs make the results of these studies difficult to generalize to all populations. In the IDEAL study, most of the patients (70%) enrolled were Caucasian, and only 9.2% were Asian. Here, we aimed to compare the survival and other clinical outcomes of patients starting dialysis for ESRD according to initiation time in a Korean prospective cohort study using propensity score-matching analysis.

## Methods

### Study Participants

We enrolled adult patients (≥20 years old) who were started on maintenance dialysis for ESRD between August 2008 and March 2013 through an ongoing cohort study (Clinical Research Center for End Stage Renal Disease, CRC for ESRD) in South Korea. The CRC for ESRD is a nationwide, multi-center, web-based, prospective cohort of CKD patients who have started dialysis (clinicaltrial.gov NCT00931970) [28]. The CRC for ESRD cohort began to register ESRD patients on dialysis in July 2008, and 31 hospitals in South Korea are currently participating. All of the patients were informed about the study and participated voluntarily with written consent. The study was approved by the institutional review board at each center. [The Catholic University of Korea, Bucheon St. Mary's Hospital; The Catholic University of Korea, Incheon St. Mary's Hospital; The Catholic University of Korea, Seoul St. Mary's Hospital; The Catholic University of Korea, St. Mary's Hospital; The Catholic University of Korea, St. Vincent's Hospital; The Catholic University of Korea, Uijeongbu St. Mary's Hospital; Cheju Halla General Hospital; Chonbuk National University Hospital; Chonnam National University Hospital; Chung-Ang University Medical Center; Chungbuk National University Hospital; Chungnam National University Hospital; Dong-A University Medical Center; Ewha Womans University Medical Center; Fatima Hospital; Gachon Medical School Gil Medical Center; Inje University Busan Paik Hospital; Kyungpook National University Hospital; Kwandong University College of Medicine, Myongji Hospital; National Health Insurance Corporation Ilsan Hospital; National Medical Center; Busan National University Hospital; Samsung Medical Center; Seoul National University Boramae Medical Center; Seoul National University Hospital; Seoul National University, Bundang Hospital; Yeungnam University Medical Center; Yonsei University, Severance Hospital; Yonsei University, Gangnam Severance Hospital; Ulsan University Hospital; Wonju Christian Hospital (in alphabetical order)]. All of the investigators conducted this study in accordance with the guidelines of the 2008 Declaration of Helsinki.

After the last enrollments in March 2013, participants were followed until October 2013 to observe at least 6-month mortality and clinical outcomes. Patients whose creatinine levels were missing at the time of dialysis initiation were excluded. Patients were categorized into the early-start group or the late-start group according to whether their eGFR was greater or less than the mean eGFR value at the start of dialysis. The eGFR was calculated using CKD-Epidemiology Collaboration (CKD-EPI) equations [29]. The modified Charlson co-morbidity index (mCCI) was calculated for each patient at the initiation of dialysis. The mCCI was developed to predict one-year mortality, and it has been validated in ESRD patients [30], [31].

### Clinical Outcomes

The primary outcome was all-cause mortality after the start of dialysis. The secondary outcomes included first hospitalization, cardiovascular events, changes in dialysis modality, vascular complications in hemodialysis patients, and peritonitis in peritoneal dialysis patients. Hospitalization was defined as admission for at least 24 hours, excluding diagnostic work-ups for transplantation. Cardiovascular events included clinical events requiring admission for ischemic heart disease, congestive heart failure, arrhythmia, or cerebrovascular disease. Changes in dialysis modality included shift from hemodialysis to peritoneal dialysis or vice versa. Vascular complications included vascular events requiring angioplasty, surgical intervention, or changes in vascular catheters for hemodialysis. Peritonitis was defined as the presence of the following conditions: 1) signs and symptoms of peritoneal inflammation; and 2) a peritoneal effluent white blood cell count greater than 100 cells/mm^3^ and a neutrophil percentage greater than 50%.

### Statistical Analysis

The propensity scores, which were calculated from the logistic regression models, represent the probability of being assigned to either an early or a late dialysis initiation. Through the matching procedure for propensity scores, the early- and late-start groups showed similar distributions of propensity scores, indicating that the differences in covariates between the two groups were minimized. We matched propensity scores one by one using nearest neighbor methods, no replacement, and 0.2 caliper width. The characteristics of both the early- and late-start groups were compared before and after propensity score matching.

Continuous variables are expressed as the mean and standard deviation, and categorical variables are presented as frequencies with percentages. Continuous variables were compared using a t-test, and categorical variables were compared using the Chi-square test or Fisher's exact tests. Survival was compared using Kaplan-Meier curve and log-rank test. IBM SPSS software (version 21.0) was used in all descriptive and survival analysis, and R software (version 2.14.2) was used in the propensity score matching. A two-tailed P value <0.05 was considered statistically significant.

## Results

### Patient Characteristics

**Figure 1:**
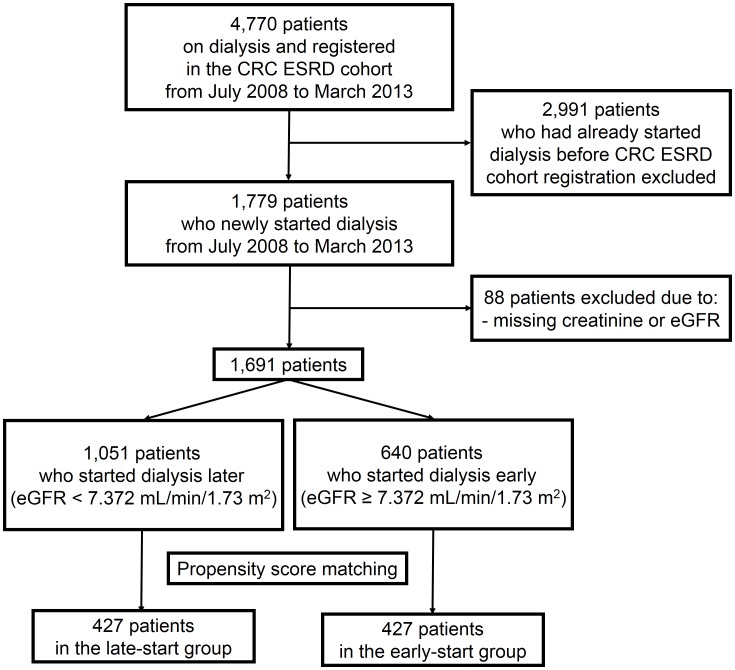
Flow chart of study enrollment. Between August 2008 and March 2013, 1069 dialysis patients with end-stage renal disease were initially enrolled. After propensity score matching, 854 patients remained in the final analysis.

Initially, among the 4770 patients retrieved from the CRC for ESRD database, 2991 dialysis patients who had started dialysis before cohort registration were excluded (Figure 1). In addition, 88 patients without information on serum creatinine levels and/or eGFR at the time of dialysis initiation were excluded from the analysis. Ultimately, a total of 1691 adult patients who started maintenance dialysis for ESRD were enrolled. Patients were classified into the early-start group (eGFR greater than the mean value) or late-start group (eGFR less than the mean value) based on the mean value of eGFR at the start of dialysis, which was 7.372 ml/min/1.73 m^2^.

The patients' clinical and laboratory characteristics are compared and summarized in Table 1. The mean age was 56.6±14.3 years old, and 61.4% of the patients were male. Patients with diabetes as a primary cause of renal disease comprised 51.2% of the study population. Most of the patients (71.1%) received hemodialysis as the modality for renal replacement therapy. Before propensity score matching, 1051 patients were in the late-start group, and 640 patients were in the early-start group. Considering all of the participants, the mean eGFR values were 11.2±8.1 ml/min/1.73 m^2^ in the early-start group and 5.0±1.4 ml/min/1.73 m^2^ in the late-start group. In the early-start group, the patients were older (58.8±14.4 vs. 55.3±14.0 years old, P<0.001), and diabetic kidney disease (59.7% vs. 46.8%) was more common. A total of 69.6% of the patients in the early-start group received hemodialysis as the first modality of dialysis, whereas 30.4% of the patients started with peritoneal dialysis. The mCCI was higher in the early-start group. Systolic and diastolic blood pressure, serum phosphorus, uric acid, albumin, intact parathyroid hormone (iPTH), and high-sensitive C-reactive protein (hsCRP) levels, use of vitamin D or phosphate binders were lower, and hemoglobin and calcium levels were higher in the early-start group. The adequacy of hemodialysis (single-pool Kt/V) was slightly lower in the early-start group of hemodialysis patients, but the weekly Kt/V of peritoneal dialysis was not different between the two groups.

**Table 1:** Patient characteristics before and after propensity score matching.

Variables	Before PSM				After PSM			
	Late Start (N = 1051)	Early Start (N = 640)	P	Standardized Difference	Late Start (N = 427)	Early Start (N = 427)	P	Standardized Difference
Age (years old)	55.3±14.0	58.8±14.4	<0.001	0.227	57.6±13.1	57.4±14.3	0.853	−0.012
Sex (male)	609 (57.9%)	430 (67.2%)	<0.001	−0.227	264 (61.8%)	272 (63.7%)	0.571	−0.040
Primary renal disease			<0.001	−0.254			0.339	−0.059
Diabetes	488 (46.8%)	377 (59.7%)			240 (56.2%)	244 (57.1%)		
Hypertension	187 (17.9%)	83 (13.2%)			57 (13.3%)	68 (15.9%)		
Glomerulonephritis	180 (17.3%)	58 (9.2%)			63 (14.8%)	44 (10.3%)		
Other	114 (10.9%)	59 (8.4%)			37 (8.7%)	38 (8.9%)		
Unknown	74 (7.1%)	54 (8.6%)			30 (7.0%)	33 (7.7%)		
Type of dialysis			0.266	0.070			0.653	−0.030
Hemodialysis	758 (72.1%)	444 (69.6%)			298 (69.8%)	304 (71.2%)		
Peritoneal dialysis	293 (27.9%)	194 (30.4%)			129 (30.2%)	123 (28.8%)		
Systolic BP (mmHg)	142.6±24.1	139.6±22.6	0.015		142.9±23.0	140.3±23.0	0.108	
Diastolic BP (mmHg)	78.9±14.8	76.3±13.6	0.001		78.2±14.5	77.4±14.0	0.409	
BMI (kg/m^2^)	23.2±3.5	23.0±3.4	0.380	−0.055	23.2±3.3	23.1±3.5	0.742	−0.023
Charlson comorbidity index	4.69±2.25	5.80±2.66	<0.001	0.402	5.27±2.29	5.32±2.53	0.734	0.021
WBC (/mm^3^)	5790±3924	5212±3956	0.004	−0.149	5319±3986	5450±3891	0.625	0.033
Hemoglobin (g/dL)	8.7±2.5	9.6±4.7	<0.001	0.178	9.1±3.3	9.2±1.5	0.510	0.023
Calcium (md/dL)	7.7±3.1	8.1±3.0	0.010	0.130	8.1±4.6	8.2±3.6	0.729	0.031
Phosphorus (md/dL)	6.2 ±2.8	4.6±2.6	<0.001	−0.597	4.9±1.3	4.8±1.3	0.209	−0.041
Uric acid (md/dL)	8.8±5.0	7.2±2.6	<0.001	−0.600	7.8 ±2.0	7.7 ±2.6	0.519	−0.040
Albumin (g/dL)	3.4±0.6	3.2±0.6	<0.001	−0.216	3.3±0.6	3.3±0.6	0.481	−0.046
Creatinine (md/dL)	10.45±5.91	5.35±1.35	<0.001		9.19±3.24	5.49±1.26	<0.001	
eGFR* (ml/min/1.73 m^2^)	5.0±1.4	11.2±8.1	<0.001		5.5±1.2	10.4±4.9	<0.001	
Ferritn (ng/ml)	308.4 ±500.2	327.6 ±401.0	0.458		281.8±431.0	318.8±372.6	0.222	
Cholesterol (mg/dL)	159.5±48.5	155.4±51.6	0.120		158.3 ±48.6	156.4±48.3	0.578	
hsCRP (mg/L)	4.0±16.4	6.9±20.5	0.004		5.9±23.5	4.4±12.4	0.261	
iPTH (pg/mL)	300.4±255.6	200.0±198.0	<0.001		269.8±228.2	210.3±210.9	0.001	
Use of vitamin D	188 (17.9%)	62 (9.7%)	<0.001		67 (15.7%)	44 (10.3%)	0.025	
Use of phosphate binders	575 (54.7%)	288 (45.0%)	<0.001		233 (54.6%)	211 (49.4%)	0.212	
Single-pool Kt/V†	1.33±0.47	1.24±0.55	0.047		1.33±0.40	1.23±0.59	0.121	
Weekly Kt/V‡	2.47±1.02	2.59±1.38	0.735		2.79±1.02	2.83±1.32	0.994	

BMI, body mass index; iPTH, intact PTH; PSM, propensity score-matching; WBC, white blood cell count.

^*^Levels of eGFR were calculated using the CKD-EPI equation.

† Single-pool Kt/V was measured in patients with hemodialysis.

‡ Weekly Kt/V was measured in patients with peritoneal dialysis.

### Propensity Matching of Cohort

**Figure 2:**
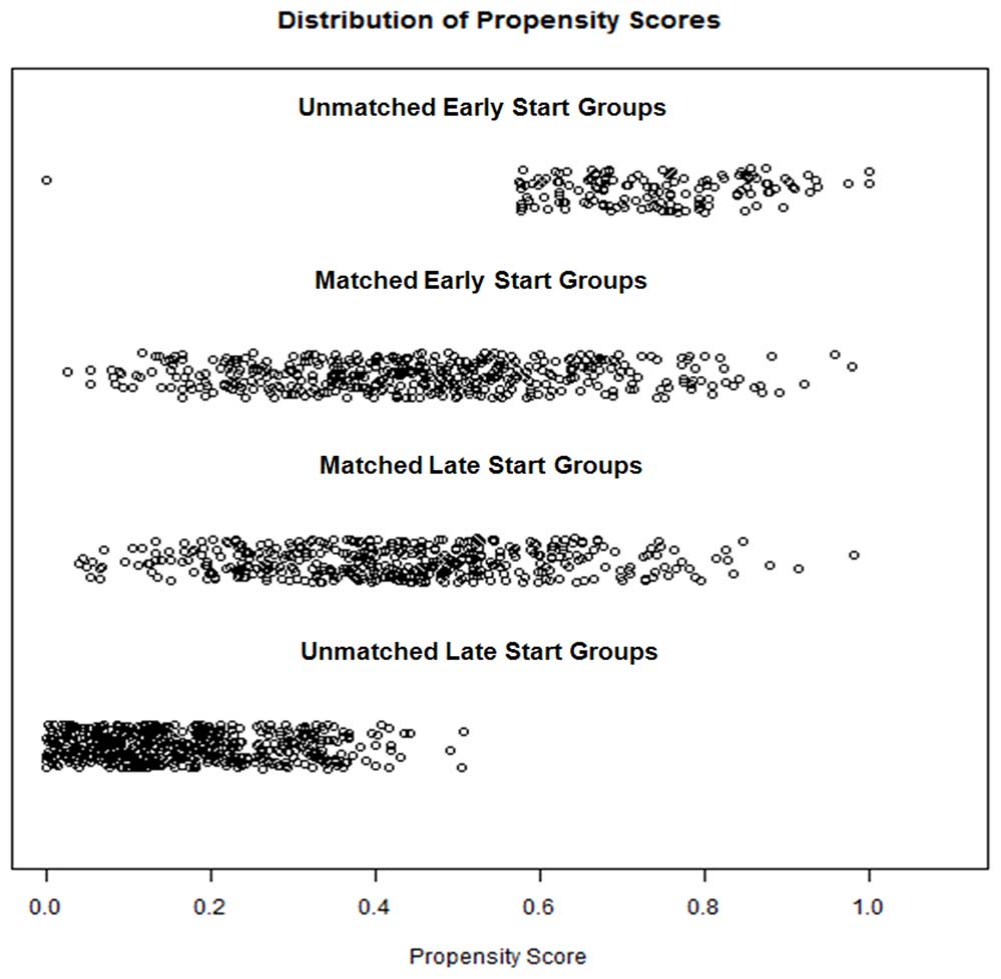
Distribution of propensity scores before and after propensity score matching. The propensity scores of unmatched patients were significantly different between the early- and late-start groups. The propensity scores of matched patients were almost the same between groups.

We performed a logistic regression analysis to obtain propensity scores for dialysis initiation timing using the following covariates: age, sex, primary renal disease, type of dialysis, body mass index, mCCI, hemoglobin level, calcium level, phosphorus level, uric acid level, albumin level, and comorbidities. After propensity score matching, 854 patients (427 in each group) remained. The distributions of propensity scores before and after matching are illustrated in Figure 2. In the propensity score-matched participants, almost all of the baseline parameters, including age, sex, primary renal disease, type of dialysis, systolic and diastolic blood pressure, body mass index, mCCI, hemoglobin levels, serum calcium, phosphorus, uric acid, glucose, albumin, hsCRP levels, and adequacy of dialysis (single-pool Kt/V in hemodialysis patients and weekly Kt/V in peritoneal dialysis patients) were similar between the groups. The propensity scores of the matched patients were almost the same between the early- and late-start groups.

### Survival and Clinical Outcomes

**Figure 3:**
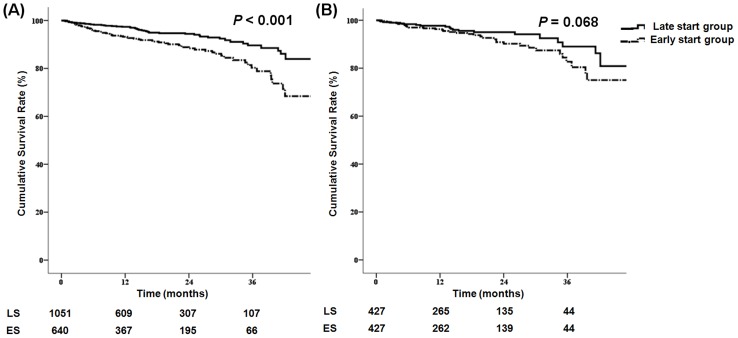
Kaplan-Meier patient survival curve for the timing of dialysis initiation. (A) Before matching, the patients in the early start group had poor survival. (B) After propensity score matching, patients in the early- and late-start groups showed no differences in survival.

The patients' survival rates are shown in Figure 3. Before propensity score matching, the early-start group had a worse survival rate (P<0.001). Hospitalization (P = 0.195), cardiovascular events (P = 0.352), vascular access complications (P = 0.158), changes in dialysis modality (P = 0.660), and peritonitis (P = 0.833) were not different between the groups (Figure S1). After matching, patient survival, hospitalization, cardiovascular events, vascular access complications, change of dialysis modality, and peritonitis were not different between the groups.

**Figure 4:**
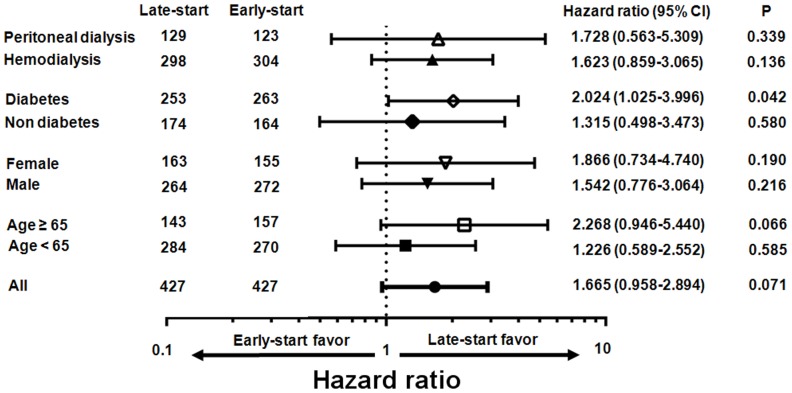
Hazard ratio (HR) for mortality of early dialysis initiation using a Cox proportional analysis in the propensity score-matched cohort. The hazard of early dialysis initiation was not elevated in any subgroup except patients with diabetes. In patients with diabetes, the hazard of early dialysis initiation was significantly greater (HR 2.024, 95% CI 1.025–3.996).

Subgroup analyses of all-cause mortality were performed in the propensity score-matched cohort, and the hazard ratio of starting dialysis early is illustrated in Figure 4 using a Cox proportional analysis. The hazard associated with starting dialysis early was not elevated in any subgroups except patients with diabetes. In patients with diabetes, the hazard associated with an early-start to dialysis was significantly greater (HR 2.024, 95% CI 1.025–3.996).

The causes of mortality are listed in Table 2. The distributions of the mortality causes were not different before (P = 0.817) and after (P = 0.521) propensity score matching. In propensity score-matched participants, mortality rates from cardiovascular events (P = 0.630), cerebrovascular accidents (P = 0.659), infections (P = 0.783), and malignancies (P = 0.984) were not different between the early- and late-start groups. Cardiovascular events, including acute myocardial infarction, arrhythmia, cardiac arrest, valvular heart disease, and congestive heart failure, were the leading causes of mortality.

**Table 2:** Causes of patient mortality.

Causes of mortality	All participants (N = 1691)			PS-matched participants (N = 854)		
	All participants	Late-start (N = 1051)	Early-start (N = 640)	PS-matched participants	Late-start (N = 427)	Early-start (N = 427)
Cardiovascular events	34 (29.6%)	17 (33.3%)	17 (26.6%)	16 (29.1%)	7 (33.3%)	9 (26.5%)
Cerebrovascular events	7 (6.1%)	2 (3.9%)	5 (7.8%)	5 (9.1%)	2 (9.5%)	3 (8.8%)
Infections	30 (26.1%)	15 (29.4%)	15 (23.4%)	13 (23.6%)	6 (28.6%)	7 (20.6%)
Malignancies	7 (6.1%)	3 (5.9%)	4 (6.3%)	2 (3.6%)	1 (4.8%)	1 (2.9%)
Other	22 (19.1%)	8 (15.7%)	14 (21.9%)	10 (18.2%)	1 (4.8%)	9 (26.5%)
Unknown	15 (13.0%)	6 (11.8%)	9 (14.1%)	9 (16.4%)	4 (19.0%)	5 (14.7%)

## Discussion

We evaluated the effects of early dialysis initiation on all-cause mortality and other clinical outcomes using a propensity score-matching analysis in an Asian prospective cohort study. Before matching, the early-start group seemed to have poorer survival than the late-start group. However, after matching, these differences in survival disappeared, and there were no significant differences in all-cause mortality or other clinical outcomes.

Comparing the entire cohort of participants, the early start-group was older than the late-start group. In addition, the proportions of male patients and cases of diabetic kidney disease were greater in the early-start group. The mCCI was also higher in the early-start group. Although systolic and diastolic blood pressure levels were lower and hemoglobin levels were higher in the early-start group, serum albumin levels were lower in the early-start group. In summary, except for residual renal function, the demographic and laboratory parameters were worse in the early-start group. It is possible that the patients in the early-start group initiated dialysis earlier because they had more complications due to ESRD. The high burden of comorbidities in the early-start group might have been the principal cause of poor survival before matching. Indeed, after adjusting for these confounding factors using propensity score-matching methods, survival and clinical outcomes were comparable between groups.

Although the proportion of early dialysis initiation remained stable between 2009 and 2011 in the United States, the percentage of patients who started dialysis early had grown steadily until 2009, up to 54% [10]. A worldwide trend toward early dialysis initiation had been supported by the belief that increased solute clearance could improve patient survival and clinical outcomes. Prolonged uremia can decrease appetite and evoke anorexia, poor oral intake, and malnutrition [32]. Several studies had shown that decreased eGFR values at the time of dialysis initiation were closely associated with poor nutritional status and mortality [33]–[37]. In addition, there had been concern that delaying dialysis might fail to prevent fatal uremic complications, including severe hyperkalemia, uncontrolled hypertension, pulmonary edema, pericarditis, and encephalopathy. In fact, before the IDEAL study, clinical guidelines had permitted early dialysis initiation for an eGFR of over 10 ml/min/1.73 m^2^ if there were relevant uremic symptoms or evidence of malnutrition [38]–[41]. In the United States, reflecting these guidelines, the mean eGFR at dialysis initiation increased from 8.1 ml/min/1.73 m^2^ in 1997 to 10.8 ml/min/1.73 m^2^ in 2007 [42]. However, the association between low eGFR at the time of dialysis initiation and poor survival cannot be used as direct evidence justifying early dialysis initiation. Almost studies reporting the benefit of early dialysis initiation were of a retrospective observational design, and the adjustments made for demographic factors and comorbid conditions were insufficient [33], [34], [43], [44]. In addition, the possibility of lead-time bias in the studies favoring early dialysis initiation should be considered. A survival benefit of early dialysis initiation could result from the statistical misinterpretation of the fact that patients had merely started dialysis early, rather than that they actually lived longer. Korevaar et al. reported that the survival benefit of 2.5 months in the patients with early dialysis initiation was overwhelmed by the effect of delaying dialysis approximately 4.1 months in the patients with late dialysis initiation [45].

Notably, contrary to general expectations, recent observational studies have shown that early dialysis initiation was irrelevant to survival benefits or even associated with poor clinical outcomes [11], [14], [26], [46]–[50]. Starting dialysis early can expose ESRD patients to dialysis-associated complications [51]. The decline in residual renal function can progress at a rapid pace, even after dialysis [52]. Dialysis therapy can also result in protein loss and aggravate nutritional status in ESRD patients [53]. Catheter- or access site-related peritoneal or bloodstream infections are increased in patients undergoing dialysis [54]. These factors can thus collectively contribute to the poor survival and negative clinical outcomes of patients with early dialysis initiation. Although there have been several lines of evidence supporting the harmful effects of early dialysis initiation, there is still a debate as to whether a high eGFR itself is the main cause of poor outcomes in patients with early dialysis initiation. Most eGFR equations are based on serum creatinine levels, so there is a possibility of overestimating the eGFR in cases of low serum creatinine levels due to low muscle mass or fluid overload [55]. Beddhu et al. reported that high eGFR values were closely associated with increased mortality, but high creatinine clearance was not [56]. In addition, survivor bias can overestimate the risk of early dialysis initiation due to the limitations of observational studies. Patients who had died before initiating dialysis were excluded from the analyses of observational studies, and the number of such patients is likely to be higher in the late-start group. Therefore, those who start dialysis later may collectively comprise a healthier group. Crew et al. minimized the lead-time and survivor bias through the enrollment of patients before the initiation of dialysis and analyzed clinical outcomes after the eGFR reached approximately 20 ml/min/1.73 m^2^. This work demonstrated that patients who initiated dialysis early or late did not exhibit differences in survival [57]. Lastly, patients who had a high burden of uremic symptoms and/or comorbidities are likely to start dialysis earlier. It is known that patients who are older, male, or who had diabetes, low body mass index, high comorbidities with cardiovascular complications, or poor functional status are likely to start dialysis earlier [48], [58]–[60]. These high risk comorbidities and demographic factors can aggravate survival and other clinical outcomes in patients with early dialysis initiation. Several investigations reported that the risk of early dialysis initiation decreased after multivariate adjustments for demographic factors, laboratory data, and comorbidities [46], [48]. Bao et al. investigated that high mortality was closely associated with frailty, including slowness or weakness, exhaustion, and low physical activity and found that the risks associated with early dialysis initiation disappeared after adjustment for frailty [61]. A recent meta-analysis showed that an early start to dialysis was associated with increased mortality and that patients with older age, diabetes, and high comorbidities seemed to start dialysis earlier [12]. The results of our study before matching are similar to the results of these studies. Patients in the early-start group of the present study were older and had more comorbidities, including diabetes. The poor survival of these patients disappeared after matching the covariates.

To date, only one randomized, controlled study has evaluated the association between dialysis initiation time and survival [15]. In the IDEAL study, Cooper et al. allocated ESRD patients to planned dialysis initiation at an eGFR greater than 10 ml/min/1.73 m^2^ or conventional dialysis initiation at an eGFR less than 7 ml/min/1.73 m^2^. There were no differences in survival, complications, or quality of life between the two groups. Although the actual difference in eGFR between the two groups was only approximately 2 ml/min/1.73 m^2^, the patients in the late-start group could begin dialysis approximately 6 months later than those in the early-start group. When the participants in the IDEAL study were analyzed separately by dialysis modality, more adverse events associated with fluids and electrolytes were observed in the late hemodialysis group, and more patients who had been randomized to peritoneal dialysis switched to hemodialysis; however, early dialysis initiation did not provide survival or other clinical benefits [62], [63]. It is difficult to generalize the results of the IDEAL study to all patients preparing for dialysis because the patients enrolled in the IDEAL study were almost all Caucasians and relatively well prepared for ESRD, with low rates of temporary vascular access and a high proportion of peritoneal dialysis. In addition, there was a high incidence of cross-over from the late dialysis group to the early dialysis group. However, the results of the IDEAL study clearly indicate that early dialysis initiation is not unconditionally beneficial for patients with ESRD and that the late initiation strategy can delay dialysis initiation for a proportion of well-prepared patients. The results of our study are consistent with those of the IDEAL study in that neither early nor late initiation of dialysis based on eGFR values was associated with mortality or clinical outcomes. Reflecting the results of recent investigations, newer guidelines recommend delaying dialysis and addressing relevant clinical signs or symptoms rather than instituting dialysis therapy solely based on eGFR values [16]–[18].

This study has several limitations. First, because this study is an observational cohort study beginning at the time of dialysis initiation, the possibility of survivor bias still exists. This factor can favor late dialysis initiation. In addition, because all patients enrolled were Asian, the results of this study cannot easily be generalized to all ESRD patients, as the prognosis and clinical outcomes of ESRD patients are well known to be closely associated with demographic factors and comorbid conditions. However, this study still has an advantage in that it is the only prospective and well-matched cohort study in Asian ESRD patients using propensity score-matching analysis. After matching, almost all variables, including age and comorbidities, became similar between the early- and late-start groups, except for variables associated with residual renal function. Because demographic factors, comorbid conditions, and laboratory parameters converged after matching, the possibility of bias, including lead-time bias, is minimized. In addition, because there was a substantial difference in eGFR between the groups (approximately 5 ml/min/1.73 m^2^), the effects of early dialysis initiation could be explored with confidence.

We evaluated the effects of dialysis initiation timing based on eGFR on clinical outcomes using propensity score-matching analysis. Early dialysis initiation did not improve patient survival or other clinical outcomes, including hospitalization, cardiovascular events, vascular access complications, changes in dialysis modality, or peritonitis. It is appears that, rather than the eGFR at the initiation timing of dialysis, a patient's health status, including age, sex, physical activity, and comorbidities, has a greater impact on clinical outcomes in patients initiating dialysis. Although the optimal time for dialysis initiation remains controversial, dialysis initiation should not be determined based on eGFR values alone. Residual renal function, comorbidities, and uremic symptoms should be considered before starting dialysis.

## Supporting Information

Figure S1 **Clinical outcomes other than patient survival according to the timing of dialysis initiation before and after propensity score matching.** A, B. hospitalization; C, D. cardiovascular events; E, F. dialysis modality change; G, H. vascular access complications in hemodialysis patients; I, J. peritonitis in peritoneal dialysis patients. A, C, E, G, I: before matching; B, D, F, H, J: after matching.
(TIF)

## References

[1] Zhang QL, Rothenbacher D (2008) Prevalence of chronic kidney disease in population-based studies: systematic review. BMC Public Health 8: 117. 18405348 10.1186/1471-2458-8-117 PMC2377260

[2] Collins AJ, Foley RN, Chavers B, Gilbertson D, Herzog C, et al (2014) US Renal Data System 2013 Annual Data Report. Am J Kidney Dis 63: A7. 24360288 10.1053/j.ajkd.2013.11.001

[3] Gansevoort RT, Correa-Rotter R, Hemmelgarn BR, Jafar TH, Heerspink HJ, et al (2013) Chronic kidney disease and cardiovascular risk: epidemiology, mechanisms, and prevention. Lancet 382: 339–352. 23727170 10.1016/S0140-6736(13)60595-4

[4] Wen CP, Cheng TY, Tsai MK, Chang YC, Chan HT, et al (2008) All-cause mortality attributable to chronic kidney disease: a prospective cohort study based on 462293 adults in Taiwan. Lancet 371: 2173–2182. 18586172 10.1016/S0140-6736(08)60952-6

[5] Turin TC, Tonelli M, Manns BJ, Ravani P, Ahmed SB, et al (2012) Chronic kidney disease and life expectancy. Nephrol Dial Transplant 27: 3182–3186. 22442392 10.1093/ndt/gfs052

[6] Chandna SM, Da Silva-Gane M, Marshall C, Warwicker P, Greenwood RN, et al (2011) Survival of elderly patients with stage 5 CKD: comparison of conservative management and renal replacement therapy. Nephrol Dial Transplant 26: 1608–1614. 21098012 10.1093/ndt/gfq630 PMC3084441

[7] Joly D, Anglicheau D, Alberti C, Nguyen AT, Touam M, et al (2003) Octogenarians reaching end-stage renal disease: cohort study of decision-making and clinical outcomes. J Am Soc Nephrol 14: 1012–1021. 12660336 10.1097/01.asn.0000054493.04151.80

[8] Carson RC, Juszczak M, Davenport A, Burns A (2009) Is maximum conservative management an equivalent treatment option to dialysis for elderly patients with significant comorbid disease? Clin J Am Soc Nephrol 4: 1611–1619. 19808244 10.2215/CJN.00510109 PMC2758251

[9] Rosansky S, Glassock RJ, Clark WF (2011) Early start of dialysis: a critical review. Clin J Am Soc Nephrol 6: 1222–1228. 21555505 10.2215/CJN.09301010

[10] Rosansky SJ, Clark WF (2013) Has the yearly increase in the renal replacement therapy population ended? J Am Soc Nephrol 24: 1367–1370. 23868925 10.1681/ASN.2013050458 PMC3752956

[11] Crews DC, Scialla JJ, Liu J, Guo H, Bandeen-Roche K, et al (2014) Predialysis health, dialysis timing, and outcomes among older United States adults. J Am Soc Nephrol 25: 370–379. 24158988 10.1681/ASN.2013050567 PMC3904572

[12] Pan Y, Xu XD, Guo LL, Cai LL, Jin HM (2012) Association of early versus late initiation of dialysis with mortality: systematic review and meta-analysis. Nephron Clin Pract 120: c121–131. 22584438 10.1159/000337572

[13] Susantitaphong P, Altamimi S, Ashkar M, Balk EM, Stel VS, et al (2012) GFR at initiation of dialysis and mortality in CKD: a meta-analysis. Am J Kidney Dis 59: 829–840. 22465328 10.1053/j.ajkd.2012.01.015 PMC3395227

[14] Stel VS, Dekker FW, Ansell D, Augustijn H, Casino FG, et al (2009) Residual renal function at the start of dialysis and clinical outcomes. Nephrol Dial Transplant 24: 3175–3182. 19515803 10.1093/ndt/gfp264

[15] Cooper BA, Branley P, Bulfone L, Collins JF, Craig JC, et al (2010) A randomized, controlled trial of early versus late initiation of dialysis. N Engl J Med 363: 609–619. 20581422 10.1056/NEJMoa1000552

[16] Nesrallah GE, Mustafa RA, Clark WF, Bass A, Barnieh L, et al (2014) Canadian Society of Nephrology 2014 clinical practice guideline for timing the initiation of chronic dialysis. CMAJ 186: 112–117. 24492525 10.1503/cmaj.130363 PMC3903737

[17] KDIGO (2013) KDIGO clinical practice guideline for the evaluation and management of chronic kidney disease. Kidney Int Suppl 3: 1–150. 10.1038/ki.2013.243 23989362

[18] Tattersall J, Dekker F, Heimburger O, Jager KJ, Lameire N, et al (2011) When to start dialysis: updated guidance following publication of the Initiating Dialysis Early and Late (IDEAL) study. Nephrol Dial Transplant 26: 2082–2086. 21551086 10.1093/ndt/gfr168

[19] Arce CM, Goldstein BA, Mitani AA, Winkelmayer WC (2013) Trends in relative mortality between Hispanic and non-Hispanic whites initiating dialysis: a retrospective study of the US Renal Data System. Am J Kidney Dis 62: 312–321. 23647836 10.1053/j.ajkd.2013.02.375 PMC4169882

[20] Derose SF, Rutkowski MP, Crooks PW, Shi JM, Wang JQ, et al (2013) Racial differences in estimated GFR decline, ESRD, and mortality in an integrated health system. Am J Kidney Dis 62: 236–244. 23499049 10.1053/j.ajkd.2013.01.019 PMC3723721

[21] Conley J, Tonelli M, Quan H, Manns BJ, Palacios-Derflingher L, et al (2012) Association between GFR, proteinuria, and adverse outcomes among White, Chinese, and South Asian individuals in Canada. Am J Kidney Dis 59: 390–399. 22115883 10.1053/j.ajkd.2011.09.022

[22] Barbour SJ, Er L, Djurdjev O, Karim M, Levin A (2010) Differences in progression of CKD and mortality amongst Caucasian, Oriental Asian and South Asian CKD patients. Nephrol Dial Transplant 25: 3663–3672. 20368302 10.1093/ndt/gfq189

[23] Chang JH, Rim MY, Sung J, Ko KP, Kim DK, et al (2012) Early start of dialysis has no survival benefit in end-stage renal disease patients. J Korean Med Sci 27: 1177–1181. 23091314 10.3346/jkms.2012.27.10.1177 PMC3468753

[24] Oh KH, Hwang YH, Cho JH, Kim M, Ju KD, et al (2012) Outcome of early initiation of peritoneal dialysis in patients with end-stage renal failure. J Korean Med Sci 27: 170–176. 22323864 10.3346/jkms.2012.27.2.170 PMC3271290

[25] Yamagata K, Nakai S, Iseki K, Tsubakihara Y (2012) Committee of Renal Data Registry of the Japanese Society for Dialysis Therapy (2012) Late dialysis start did not affect long-term outcome in Japanese dialysis patients: long-term prognosis from Japanese Society for Dialysis Therapy Registry. Ther Apher Dial 16: 111–120. 22458388 10.1111/j.1744-9987.2011.01052.x

[26] Hwang SJ, Yang WC, Lin MY, Mau LW, Chen HC (2010) Impact of the clinical conditions at dialysis initiation on mortality in incident haemodialysis patients: a national cohort study in Taiwan. Nephrol Dial Transplant 25: 2616–2624. 20519231 10.1093/ndt/gfq308

[27] Shiao CC, Huang JW, Chien KL, Chuang HF, Chen YM, et al (2008) Early initiation of dialysis and late implantation of catheters adversely affect outcomes of patients on chronic peritoneal dialysis. Perit Dial Int 28: 73–81. 18178951

[28] Kim do H, Kim M, Kim H, Kim YL, Kang SW, et al (2013) Early referral to a nephrologist improved patient survival: prospective cohort study for end-stage renal disease in Korea. PLoS One 8: e55323. 23372849 10.1371/journal.pone.0055323 PMC3555934

[29] Levey AS, Stevens LA, Schmid CH, Zhang YL, Castro AF 3rd, et al (2009) A new equation to estimate glomerular filtration rate. Ann Intern Med 150: 604–612. 19414839 10.7326/0003-4819-150-9-200905050-00006 PMC2763564

[30] Charlson ME, Pompei P, Ales KL, MacKenzie CR (1987) A new method of classifying prognostic comorbidity in longitudinal studies: development and validation. J Chronic Dis 40: 373–383. 3558716 10.1016/0021-9681(87)90171-8

[31] Hemmelgarn BR, Manns BJ, Quan H, Ghali WA (2003) Adapting the Charlson Comorbidity Index for use in patients with ESRD. Am J Kidney Dis 42: 125–132. 12830464 10.1016/s0272-6386(03)00415-3

[32] Fouque D, Kalantar-Zadeh K, Kopple J, Cano N, Chauveau P, et al (2008) A proposed nomenclature and diagnostic criteria for protein-energy wasting in acute and chronic kidney disease. Kidney Int 73: 391–398. 18094682 10.1038/sj.ki.5002585

[33] Liu H, Peng Y, Liu F, Xiao H, Chen X, et al (2008) Renal function and serum albumin at the start of dialysis in 514 Chinese ESRD in-patients. Ren Fail 30: 685–690. 18704816 10.1080/08860220802212619

[34] Cooper BA, Aslani A, Ryan M, Ibels LS, Pollock CA (2003) Nutritional state correlates with renal function at the start of dialysis. Perit Dial Int 23: 291–295. 12938832

[35] Pupim LB, Kent P, Caglar K, Shyr Y, Hakim RM, et al (2002) Improvement in nutritional parameters after initiation of chronic hemodialysis. Am J Kidney Dis 40: 143–151. 12087572 10.1053/ajkd.2002.33923

[36] Churchill DN (1997) An evidence-based approach to earlier initiation of dialysis. Am J Kidney Dis 30: 899–906. 9398139 10.1016/s0272-6386(97)90102-5

[37] Hakim RM, Lazarus JM (1995) Initiation of dialysis. J Am Soc Nephrol 6: 1319–1328. 8589305 10.1681/ASN.V651319

[38] Hemodialysis Adequacy 2006 Work Group (2006) Clinical practice guidelines for hemodialysis adequacy, update 2006. Am J Kidney Dis 48 Suppl 1S2–90. 16813990 10.1053/j.ajkd.2006.03.051

[39] Peritoneal Dialysis Adequacy Work Group (2006) Clinical practice guidelines for peritoneal dialysis adequacy. Am J Kidney Dis 48 Suppl 1S98–129. 16813998 10.1053/j.ajkd.2006.04.006

[40] Kelly J, Stanley M, Harris D, Caring for Australians with Renal Impairment (2005) The CARI guidelines. Acceptance into dialysis guidelines. Nephrology (Carlton) 10 Suppl 4S46–60. 16221124 10.1111/j.1440-1797.2005.00486_1.x

[41] European Best Practice Guidelines Expert Group on Hemodialysis (2002) Section I. Measurement of renal function, when to refer and when to start dialysis. Nephrol Dial Transplant 17 Suppl 77–15. 10.1093/ndt/17.suppl_7.7 12386205

[42] O′Hare AM, Choi AI, Boscardin WJ, Clinton WL, Zawadzki I, et al (2011) Trends in timing of initiation of chronic dialysis in the United States. Arch Intern Med 171: 1663–1669. 21987197 10.1001/archinternmed.2011.436 PMC8117168

[43] Traynor JP, Simpson K, Geddes CC, Deighan CJ, Fox JG (2002) Early initiation of dialysis fails to prolong survival in patients with end-stage renal failure. J Am Soc Nephrol 13: 2125–2132. 12138145 10.1097/01.asn.0000025294.40179.e8

[44] Tang SC, Ho YW, Tang AW, Cheng YY, Chiu FH, et al (2007) Delaying initiation of dialysis till symptomatic uraemia–is it too late? Nephrol Dial Transplant 22: 1926–1932. 17400562 10.1093/ndt/gfm109

[45] Korevaar JC, Jansen MA, Dekker FW, Jager KJ, Boeschoten EW, et al (2001) When to initiate dialysis: effect of proposed US guidelines on survival. Lancet 358: 1046–1050. 11589934 10.1016/S0140-6736(01)06180-3

[46] Clark WF, Na Y, Rosansky SJ, Sontrop JM, Macnab JJ, et al (2011) Association between estimated glomerular filtration rate at initiation of dialysis and mortality. CMAJ 183: 47–53. 21135082 10.1503/cmaj.100349 PMC3017253

[47] Rosansky SJ, Eggers P, Jackson K, Glassock R, Clark WF (2011) Early start of hemodialysis may be harmful. Arch Intern Med 171: 396–403. 21059968 10.1001/archinternmed.2010.415

[48] Lassalle M, Labeeuw M, Frimat L, Villar E, Joyeux V, et al (2010) Age and comorbidity may explain the paradoxical association of an early dialysis start with poor survival. Kidney Int 77: 700–707. 20147886 10.1038/ki.2010.14

[49] Sawhney S, Djurdjev O, Simpson K, Macleod A, Levin A (2009) Survival and dialysis initiation: comparing British Columbia and Scotland registries. Nephrol Dial Transplant 24: 3186–3192. 19390120 10.1093/ndt/gfp189

[50] Kazmi WH, Gilbertson DT, Obrador GT, Guo H, Pereira BJ, et al (2005) Effect of comorbidity on the increased mortality associated with early initiation of dialysis. Am J Kidney Dis 46: 887–896. 16253729 10.1053/j.ajkd.2005.08.005

[51] Wright S, Klausner D, Baird B, Williams ME, Steinman T, et al (2010) Timing of dialysis initiation and survival in ESRD. Clin J Am Soc Nephrol 5: 1828–1835. 20634325 10.2215/CJN.06230909 PMC2974384

[52] Jansen MA, Hart AA, Korevaar JC, Dekker FW, Boeschoten EW, et al (2002) Predictors of the rate of decline of residual renal function in incident dialysis patients. Kidney Int 62: 1046–1053. 12164889 10.1046/j.1523-1755.2002.00505.x

[53] Mehrotra R, Duong U, Jiwakanon S, Kovesdy CP, Moran J, et al (2011) Serum albumin as a predictor of mortality in peritoneal dialysis: comparisons with hemodialysis. Am J Kidney Dis 58: 418–428. 21601335 10.1053/j.ajkd.2011.03.018 PMC3159826

[54] Nguyen DB, Lessa FC, Belflower R, Mu Y, Wise M, et al (2013) Invasive methicillin-resistant Staphylococcus aureus infections among patients on chronic dialysis in the United States, 2005–2011. Clin Infect Dis 57: 1393–1400. 23964088 10.1093/cid/cit546 PMC3805174

[55] Grootendorst DC, Michels WM, Richardson JD, Jager KJ, Boeschoten EW, et al (2011) The MDRD formula does not reflect GFR in ESRD patients. Nephrol Dial Transplant 26: 1932–1937. 21056944 10.1093/ndt/gfq667

[56] Beddhu S, Samore MH, Roberts MS, Stoddard GJ, Ramkumar N, et al (2003) Impact of timing of initiation of dialysis on mortality. J Am Soc Nephrol 14: 2305–2312. 12937307 10.1097/01.asn.0000080184.67406.11

[57] Crews DC, Scialla JJ, Boulware LE, Navaneethan SD, Nally JV Jr, et al (2014) Comparative effectiveness of early versus conventional timing of dialysis initiation in advanced CKD. Am J Kidney Dis 63: 806–815. 24508475 10.1053/j.ajkd.2013.12.010 PMC4117406

[58] van de Luijtgaarden MW, Noordzij M, Tomson C, Couchoud C, Cancarini G, et al (2012) Factors influencing the decision to start renal replacement therapy: results of a survey among European nephrologists. Am J Kidney Dis 60: 940–948. 22921638 10.1053/j.ajkd.2012.07.015

[59] Streja E, Nicholas SB, Norris KC (2013) Controversies in timing of dialysis initiation and the role of race and demographics. Semin Dial 26: 658–666. 24102770 10.1111/sdi.12130 PMC3836868

[60] Slinin Y, Guo H, Li S, Liu J, Morgan B, et al (2014) Provider and care characteristics associated with timing of dialysis initiation. Clin J Am Soc Nephrol 9: 310–317. 24436477 10.2215/CJN.04190413 PMC3913233

[61] Bao Y, Dalrymple L, Chertow GM, Kaysen GA, Johansen KL (2012) Frailty, dialysis initiation, and mortality in end-stage renal disease. Arch Intern Med 172: 1071–1077. 22733312 10.1001/archinternmed.2012.3020 PMC4117243

[62] Collins J, Cooper B, Branley P, Bulfone L, Craig J, et al (2011) Outcomes of patients with planned initiation of hemodialysis in the IDEAL trial. Contrib Nephrol 171: 1–9. 21625083 10.1159/000327146

[63] Johnson DW, Wong MG, Cooper BA, Branley P, Bulfone L, et al (2012) Effect of timing of dialysis commencement on clinical outcomes of patients with planned initiation of peritoneal dialysis in the IDEAL trial. Perit Dial Int 32: 595–604. 23212859 10.3747/pdi.2012.00046 PMC3524893

