# Supplementary figures and images for: Effect of Dialysis Initiation Timing on Clinical Outcomes: A Propensity-Matched Analysis of a Prospective Cohort Study in Korea
Source: PLoS One. 2014 Aug 19;9(8):e105532. doi: 10.1371/journal.pone.0105532 (PMC4138196; doi:10.1371/journal.pone.0105532)

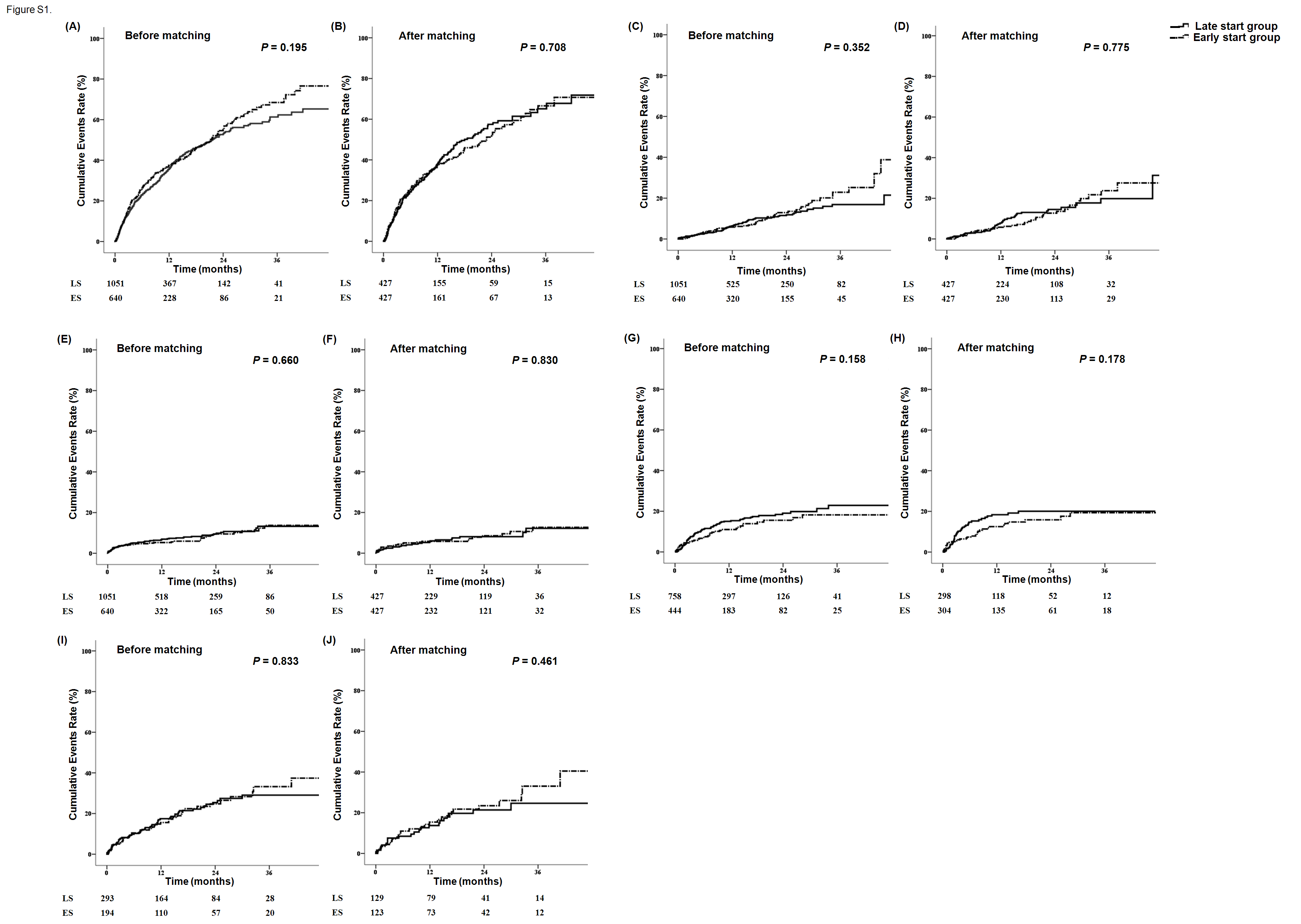

Supplement: Figure S1 — Clinical outcomes other than patient survival according to the timing of dialysis initiation before and after propensity score matching. A, B. hospitalization; C, D. cardiovascular events; E, F. dialysis modality change; G, H. vascular access complications in hemodialysis patients; I, J. peritonitis in peritoneal dialysis patients. A, C, E, G, I: before matching; B, D, F, H, J: after matching. (TIF) [file pone.0105532.s001.tif]
